# Is complex fault zone behaviour a reflection of rheological heterogeneity?

**DOI:** 10.1098/rsta.2019.0421

**Published:** 2021-02-01

**Authors:** Å. Fagereng, A. Beall

**Affiliations:** School of Earth and Ocean Sciences, Cardiff University, Park Place, Cardiff CF10 3AT, UK

**Keywords:** faults, shear zones, rheology, earthquakes, creep

## Abstract

Fault slip speeds range from steady plate boundary creep through to earthquake slip. Geological descriptions of faults range from localized displacement on one or more discrete planes, through to distributed shearing flow in tabular zones of finite thickness, indicating a large range of possible strain rates in natural faults. We review geological observations and analyse numerical models of two-phase shear zones to discuss the degree and distribution of fault zone heterogeneity and effects on active fault slip style. There must be certain conditions that produce earthquakes, creep and slip at intermediate velocities. Because intermediate slip styles occur over large ranges in temperature, the controlling conditions must be effects of fault properties and/or other dynamic variables. We suggest that the ratio of bulk driving stress to frictional yield strength, and viscosity contrasts within the fault zone, are critical factors. While earthquake nucleation requires the frictional yield to be reached, steady viscous flow requires conditions far from the frictional yield. Intermediate slip speeds may arise when driving stress is sufficient to nucleate local frictional failure by stress amplification, or local frictional yield is lowered by fluid pressure, but such failure is spatially limited by surrounding shear zone stress heterogeneity.

This article is part of a discussion meeting issue ‘Understanding earthquakes using the geological record’.

## 1. Introduction

Faults are classically thought to creep steadily or slip episodically in earthquakes, with more complex conceptual models involving seismogenic patches embedded within otherwise aseismic faults [1–4]. It is, however, now clear that faults slip at a continuum of speeds [5]. Here, we review and discuss this slip rate continuum in the context of geological fault zone structure.

## 2. Depth-dependency of fault zone structure, strength and frictional stability

**Figure 1.**
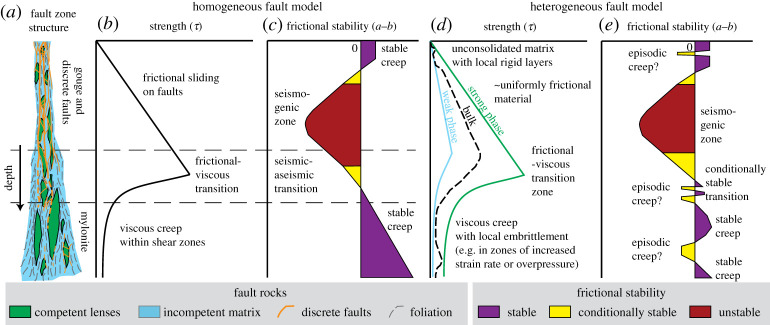
The generalized structural model of a fault zone, here shown for a vertical fault (*a*), illustrates a dominantly brittle regime of discrete faults within a wider damage zone underlain by a dominantly ductile regime where shear is accommodated within mylonites [6]. In (*b*), the same brittle–ductile two-layer system is described by a shallower layer where frictional strength increases with depth, down to a frictional–viscous transition where viscous deformation becomes easier than frictional failure and strength decreases with increased temperature and pressure [7]. (*c*) Illustrates that the transition between shallow frictional failure and deeper viscous creep may also be comparable to depth-dependent changes in steady-state frictional stability [9]. The models in (*b*) and (*c*) are simplified, and assume a homogeneous lithology subject to constant stress and strain rate. When more complexity is included, variations thereof are more appropriate, as shown by examples in (*d*) and (*e*). (Online version in colour.)

A now classic model of fault zone structure describes an upper brittle zone governed by pressure-dependent frictional sliding above a deeper ductile zone with grain-size and/or temperature-dependent viscous rheology [6–8] (figure 1*a*,*b*). Geologically, the upper, frictional regime is characterized by brittle fault rocks, including gouges, cataclasites and pseudotachylytes (lithified friction melt), where one or more discrete fault cores are surrounded by a fractured damage zone [2,10]. The deeper, viscous regime is characterized by mylonites, defined as recrystallized and/or neocrystallized rocks, commonly with relatively thin (cm-m), anastomosing higher strain zones within a broad (>km) foliated shear zone [11–13]. The shallower and deeper fault rocks reflect a well-recognized crustal strength profile, where frictional strength increases with increasing normal stress, and viscous strength decreases with increasing temperature [7]. The frictional–viscous transition is defined where the shear stresses (*τ*) driving frictional and viscous deformation are equal for some given strain-rate, composition, stress regime and thermal gradient. In reality, this depth is going to be a broad zone where both frictional and viscous deformation mechanisms are active [14,15]. Note that we have here defined ‘brittle’ and ‘ductile’ as descriptive terms for macroscopically localized and distributed deformation, respectively. ‘Frictional’ and ‘viscous’, respectively, refer to mechanisms where shear stress is proportional to normal stress and shear strain rate. We avoid the term ‘plastic’, but recognize that what we describe as ‘viscous’ deformation will have an activation energy.

The geological and mechanical observations outlined above for a homogeneous fault zone relate to, but do not fully describe, the fault’s depth-dependent seismic behaviour. This is further described by velocity-dependence of friction, denoted by the parameter (*a* − *b*), defined by $\tau =\sigma_{n}^{\prime }[\mu^{*}+(a-b)\log(V/V^{*})]$ in the steady-state form of the rate and state friction law [9,16–20] (figure 1*c*). Here, $\mu^{*}$ is the coefficient of friction at reference velocity $V^{*}$, *σ*_*n*_′ is effective normal stress and *V* is slip velocity. In this framework, velocity-strengthening faults (where (*a* − *b*) > 0) accommodate displacement by stable, steady sliding, because slip does not accelerate after nucleation. Note that (*a* − *b*) can vary with velocity [21–24], and evolve as fault rocks develop [25,26], and ruptures can therefore propagate within steady-state stable regimes under some conditions [27–29]. Velocity-weakening faults (where (*a* − *b*) < 0) are unstable if the critical stiffness (*k*_*c*_), a threshold that depends on material properties and effective normal stress, exceeds the system stiffness (*k*), determined by the wallrock stiffness. Unstable fault patches can generate earthquakes under static loading. Conditional stability occurs when (*a* − *b*) < 0 and 0 < *k*_*c*_ < *k*. Note that *k* decreases as a slipping region grows, such that conditionally stable fault patches can be destabilized as they reach a critical length scale known as the nucleation length [9]. Conditionally stable areas cannot nucleate earthquakes without a dynamic load, but earthquakes may propagate into a conditionally stable field if the dynamic velocity step is sufficient. This framework infers that rocks in the deep viscous regime are velocity-strengthening because of the stable sliding observed there, and predicts that stable sliding prevails at very shallow depths where poorly lithified rocks accommodate displacement by granular flow involving dilatancy-hardening [30]. The seismogenic zone, where earthquakes can nucleate, covers a depth range between these shallow and deep aseismic, stably sliding zones. Several experiments have correlated the seismogenic zone, defined as where steady-state frictional sliding is potentially unstable, with temperatures in the range $100\text{--}350^{\circ }\text{C}$ in quartzofelspathic rocks [31] and phyllosilicate-rich gouges [32,33]; similar to thermal constraints determined from combining thermal models and the depth-distribution of seismicity [34,35]. The seismogenic zone is considered separated from the stably sliding zones above and below by transitional regimes of conditional stability [9,19] (figure 1*c*).

In transitional, conditionally stable regimes, slip speeds intermediate between steady creep and earthquakes have now been observed globally in ‘slow earthquakes’, a term that includes slow slip and low frequency earthquakes. ‘Slow slip events’ (SSEs) have been observed geodetically in well-instrumented plate boundary zones across the globe over the last two decades, and represent episodes of transient creep that repeat near-periodically at rates of mm to cm per day [36,37]. Low frequency earthquakes (LFEs) are energetic pulses depleted in high frequencies, relative to ordinary earthquakes, and represent plate boundary shear displacement [38,39]. LFEs are commonly spatially and temporally associated with SSEs in ‘episodic tremor and slip’ (ETS) events, when slow slip is accompanied by persistent, low frequency seismic signals that comprise swarms of LFEs [40–42]. Slow earthquakes have now been observed at depths from no more than a few kilometres below the sea floor in Japan and New Zealand [43,44], to as much as 90 km in the Alaskan subduction zone [45]. Thus, there is no distinct pressure (*P*) or *T* regime associated with slow earthquakes, but they do seem associated with conditional stability at or close to the seismic–aseismic transition [20,46,47].

## 3. Geological observations of fault zone heterogeneity

Generalized, 1-D homogeneous fault models (figure 1*a*–*c*) assume a single composition with a single depth-dependent rheology. Our aim here is to analyse the effect of rheological heterogeneity on the bulk behaviour of fault and shear zones—with focus on how strength contrasts between coexisting materials determine the depth range of the frictional–viscous transition, and potential consequences for seismic, aseismic, and intermediate behaviours.

### (a) Fault zone heterogeneity at shallow depths

**Figure 2.**
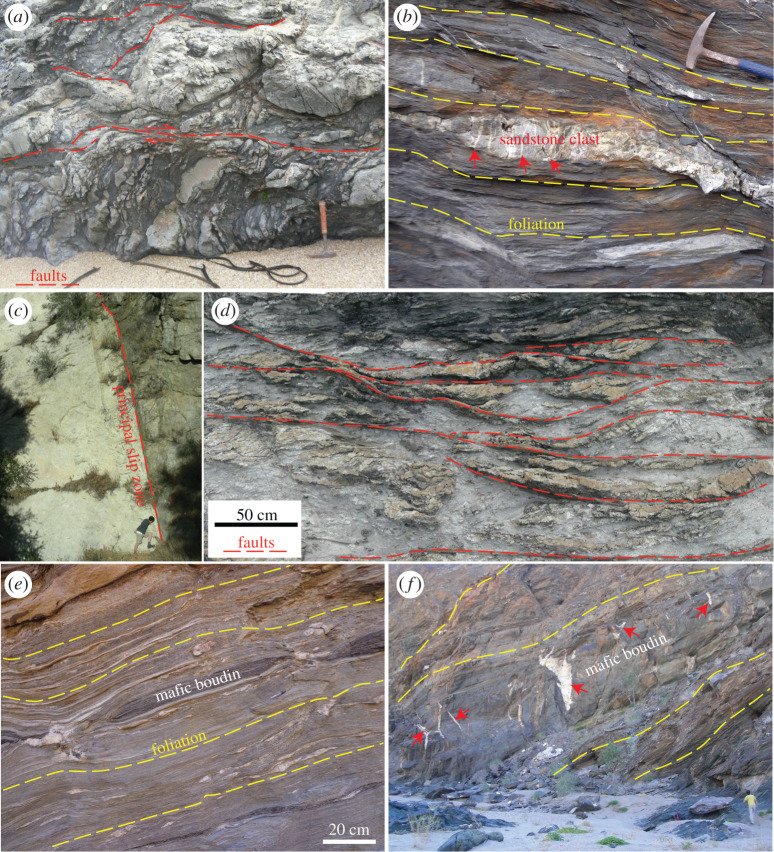
Photographs illustrating examples of lithological heterogeneity linked to deformation style, at a range of depths. In (*a*) dismembered bedding, developed by distributed independent particle flow in poorly to un-consolidated sediments, is crosscut by a subhorizontal through-going fault (Chrystalls Beach Complex, New Zealand [50]); (*b*) shows an example of a sandstone layer welded by quartz-filled fractures (red arrows at examples) that do not continue into the surrounding foliated mudstone matrix (Makimine Mélange, Kyushu, Japan [51]). An example of a cm-thick, principal slip zone in crystalline rock is found in the San Gabriel fault, California (*c*), and contrasts with an anastomosing fault system in the Chrystalls Beach Complex (*d*). In shear zones exhumed from depths below the seismogenic zone, here the Kuiseb schist that deformed at temperatures in excess of 500^°^C [52,53], competent mafic layers are boudinaged (*e*) and locally fractured and faulted (red arrows in *f* ). (Online version in colour.)

Subduction-related mélanges commonly preserve evidence for early, soft sediment deformation structures overprinted by later faults and fractures [48,49] (figure 2*a*). A common interpretation is therefore that subduction plate interface deformation style progressively changes from near-trench distributed, ductile deformation of unconsolidated sediments to localized, frictional sliding along faults in sedimentary rocks as the strata consolidate and lithify with progressive displacement and burial [50,54–56]. Accordingly, a shallow updip limit of earthquake nucleation in subduction zones has been associated with low-temperature diagenetic processes [57,58]. The transition from macroscopically ductile to brittle deformation occurs, however, at different conditions in different materials. This is clear where sandstone boudins contain fractures that do not continue into surrounding mudstone matrix (figure 2*b*). There is therefore a depth range, between shallow unconsolidated sediments and deeper consolidated rocks, where variable states of consolidation is an important source of heterogeneity.

Both strike-slip faults and subduction-related thrusts show variable sliding characteristics and heterogeneous interseismic accumulation of elastic strain at shallow depths [43,44,59–61]. Like the progressive and potentially heterogeneous consolidation observed in exhumed mélanges (figure 2*a*,*b*), the geophysical observations in active faults question the model of uniformly velocity-strengthening, unconsolidated sediments (figure 1*c*). An adaptation of the conceptual model is that shallow instabilities can be generated by one or more layers of frictionally unstable material within the frictionally stable zone (figure 1*e*). Such layers have been inferred to generate episodic creep in southern California, where relatively strong shallow intrusions and more lithified strata are inferred within otherwise unconsolidated or clay-rich velocity-strengthening fault rocks [62] (figure 1*d*,*e*). Similarly, in the Hikurangi subduction margin, highly variable input sediments are hypothesized to play a part in shallow SSEs [63,64].

That fractures are limited to a single material (as in figure 2*b*) implies that the brittle yield is not met on both sides of the bimaterial interface. Lack of fracture propagation from one material to another also implies that the static stress drop in the ductilely deformed matrix (potentially lowered by a viscously relaxed background stress) was insufficent to overcome frictional resistance, such that unstable slip was limited in dimension by the extent of relatively rigid materials. Fractures may also have been arrested prior to reaching instability if they are constrained to blocks that are smaller than a critical length scale [65]. Where shallow subduction-related thrusts have been drilled and sampled, there is evidence for both brittle and ductile deformation [66–68], also where lithological variation is not obvious [69]. Thus, local variation in deformation mode can occur without compositional heterogeneity, for example in conditionally stable regions that, over time, experience both unstable and stable deformation, because other factors, such as porosity or fluid pressure, can vary in time and space and affect frictional stability [70–72].

### (b) Heterogeneity in the seismogenic zone

The earthquake cycle has long been recognized as resulting from elastic rebound [73], where a new or existing fault accommodates stick-slip motion [74]. In this conceptual model, failure occurs when local fault-parallel shear stress, resulting from wall rock elastic strain, exceeds the cohesion plus frictional resistance of the fault rocks. Numerical models of this process are limited by computational constraints, and faults are therefore commonly considered as planar, discrete surfaces [75,76]. In such models, each point on the fault is given a single set of parameters that represents that location in time and space. This is an appropriate representation of relatively thin, planar faults [10] (e.g. figure 2*c*), where slip is well approximated by lateral interaction between segments of a single continuous fault. However, many fault zones are volumetric features [77], where fault-normal heterogeneities and interactions must also be considered.

Tabular faults that contain one or more discrete fault planes in a broad zone of foliated, viscously deforming matrix can, on the other hand, deform by a combination of distributed viscous flow of the matrix and localized frictional failure along or within more competent lenses [2,78] (e.g. figure 2*d*). In this scenario, viscous creep accommodates finite strain at slow strain rates over long time scales, whereas frictional failure typically occurs at episodically high strain rates over short time scales once a critical level of stress has been reached [79,80], although some phyllosilicates may also slide stably and frictionally at low strain rates [81]. The viscous mechanism is commonly pressure solution [80,82–84], and the rate of pressure solution will tend to vary along the fault, as it is a function of grain size, shear zone thickness, composition and other factors affecting chemical potential gradients [84–87]. Consequently, the displacement accommodated by pressure solution varies in space, resulting in along-strike and down-dip spatio-temporal variation in elastic strain energy stored in the wall rocks. Therefore, the ratio of shear stress to bulk shear zone frictional yield strength will vary spatiotemporally both along-strike and down-dip.

In two dimensions (map-view) the seismogenic zone of a rheologically heterogeneous fault may be best represented by locked patches, which creep inefficiently, in a dominantly creeping fault zone [1,3]. One may expect locked areas to dominate (by area) in localized faults lacking evidence for a wider creeping zone (e.g. figure 2*c*) and creep or mixed behaviour to be common in thicker heterogeneous fault zones with scattered rigid inclusions (e.g. figure 2*d*). The bulk fault strength of the latter will depend on the volume fractions and geometry of two (or more) rheological components [77] (figure 1*d*). For fault patches where interseismic creep is too slow to accommodate tectonically imposed displacement rates, elastic strain will accumulate until failure occurs—at a strength determined by the frictional yield of locked or partially locked areas, and potentially highly influenced by fluid pressure.

### (c) Heterogeneity below the seismogenic zone

Mylonites formed deeper than the seismogenic zone are typically characterized by structures formed by distributed flow in polyphase materials, where variable viscosity is commonly illustrated by pinch-and-swell or boudinage of relatively competent layers [88–90] (figure 2*e*). Some relatively rigid lenses and/or layers may remain in a frictional regime while adjacent rocks deform by diffusion or dislocation creep [91,92] (figure 2*f* ), potentially leading to an intermingling of velocity-weakening and velocity-strengthening rocks [77,93].

Progressive shear in a dominantly viscous shear zone may gradually increase stress in and around rigid bodies embedded within it, allowing a frictional yield to be reached locally and transiently [94,95]. Such frictional failure may be damped by surrounding viscous matrix [24,96,97], although this matrix may host dynamic rupture propagation if a critical stress level is reached [29]. Heterogeneity in viscous flow will tend to decrease with depth, theoretically, as most materials weaken exponentially with increasing temperature (figure 1*d*). Although, local variations in metamorphic reaction progress may also increase heterogeneity at depth, either by producing stronger products along a prograde path, for example in eclogitisation, [98], or by retrograde growth of weak phyllosilicates [82,99].

Whereas viscosity contrasts are likely to exist throughout shear zones accommodating dominantly stable sliding below the seismogenic zone, there are areas where the contrasts are larger. These are regions where matrix viscosity is low, creep in competent materials is slow and bulk shear stress is well below the frictional yield strength of the stronger components. Locally, however, frictional failure may occur within more competent rocks because yield strength is lowered by high fluid pressures [100], or because stress amplification occurs by loading of rigid clasts by surrounding flowing materials [101]. This local, and probably small displacement, frictional failure within otherwise viscously creeping shear zones has been invoked to explain both regular (but deep) earthquakes [94,95] and slow earthquakes [53,97,102].

## 4. Depth-dependence of viscosity contrasts in heterogeneous faults

**Figure 3.**
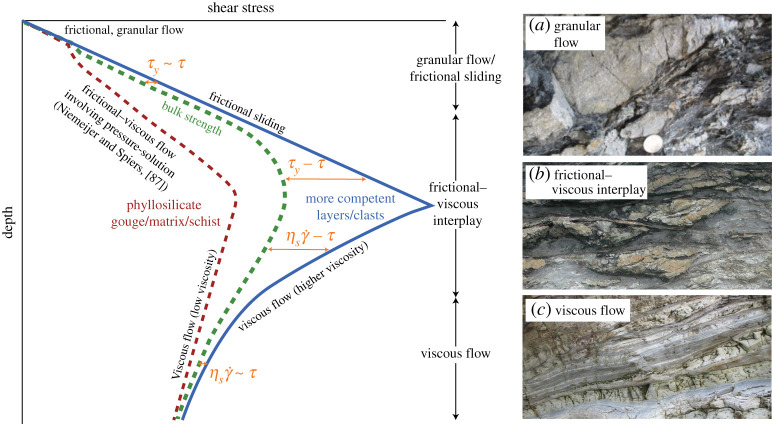
Schematic diagram showing the difference in depth-dependent strength between two components. The red, dashed curve shows a relatively weak, fine-grained phyllosilicate-rich matrix deforming preferentially by pressure solution creep. The blue, solid line represents coarser-grained layers, possibly dismembered into oblate clasts, that are too coarse for efficient pressure solution. The stress driving bulk deformation (intermediate, dashed green curve) is expected to be similar to the frictional yield strength (*τ* ∼ *τ*_*y*_) in the frictional regime, and similar to the viscous strength of competent material at the imposed strain rate ($\tau ∼\eta_{s}\dot{\gamma }$) in the viscous regime. Deviations from these conditions are associated with the frictional–viscous interplay zone. Photographs to the right show potential structures developing in this material mixture: (*a*) sandy layers dismembered by granular flow in a mudstone matrix (Makimine Mélange, Japan), (*b*) dismembered sand and chert layers in a cleaved, phyllosilicate rich matrix, cross-cut by anastomosing faults (Chrystalls Beach Complex, New Zealand), (*c*) layers of sheared metapelite, metapsammite, and metabasalt, with near-planar margins indicating small viscosity contrast (Makimine Mélange, Japan).(Online version in colour.)

Our review so far highlights various sources and degrees of rheological heterogeneity as a function of depth (figure 3). From here, we will simplify the discussion by considering fault zones as containing two components—one that is less competent and deforms efficiently by distributed flow, and another that is more competent, rigid, deforms inefficiently by viscous flow and therefore tends to fracture or host frictional failure along faults. The term ‘competent’ derives from a structural geology field term, and describes the inferred rigidity of a material at the time of deformation. The two contrasting fault zone materials can therefore be thought of as having low (*η*_*w*_) and high (*η*_*s*_) viscosities at a steady creep rate, and an effective bulk viscosity describing the combined visco-brittle deformation, defined by $\eta^{\prime }=\tau /\dot{\gamma }$ where $\dot{\gamma }$ is shear strain rate, itself defined by slip velocity divided by deforming thickness (*v*/*w*).

We consider a low viscosity, phyllosilicate-rich fault zone containing rigid lenses (figure 3), as typical of many fault zones as discussed above. The competent component has a frictional yield strength that increases linearly with depth, from the surface to a depth where viscous creep can occur at the imposed strain rate. This linear increase in frictional strength does not occur where the fluid pressure follows a lithostatic gradient below a fluid retention depth; in such examples strength can be uniform and low at depths of fluid overpressure [103,104]. Conceptually, this is no different from ‘Christmas Tree’ strength profiles discussed by many authors [7]. The less competent material represents phyllosilicate-rich fault gouges, foliated matrix material in tabular fault zones, and the least competent element of micaceous schists. The plot in figure 3 is intentionally plotted with no scale, as the absolute values of shear strengths and depths will depend on rocktypes (both composition and grain size), thermal gradient, tectonic regime, strain rate, fluid pressure and other parameters. We use this conceptual plot to define three conceptual regimes.

### (a) Granular flow and/or frictional sliding

At the shallowest depths the rheology is granular flow and/or frictional sliding, with possible effect of pressure solution. In sheared sedimentary sequences, the typical observation is dismembered sandy layers in a muddier matrix (figure 3*a*); however, one can think more generally of stronger layers dismembered in a weaker matrix, where strength is defined as the frictional yield *τ*_*y*_ = *C*_0_ + *μ*(*σ*_*n*_ − *P*_*f*_). Here, *C*_0_ is cohesion and *μ* is static coefficient of friction, such that both level of consolidation/cementation and frictional properties determine relative strengths. This means that both composition and consolidation state is important, and relative strength may vary, and locally invert, as sedimentary layers undergo diagenesis at different rates [105]. Friction is typically velocity-strengthening at these shallow depths [30,31,106].

### (b) Frictional–viscous interplay

At depths where rocks are consolidated, faulting is typically governed by some combination of pressure-solution and frictional sliding [80,86,87,107]. The conceptual strength curve for phyllosilicate-rich gouges (figure 3) follows a frictional–viscous flow for a micaceous matrix with soluble, rigid inclusions [86,87]. Deeper than a depth determined by the temperature where pressure solution becomes effective, roughly $100\text{--}150^{\circ }$C for quartz [108,109], the phyllosilicate-rich gouge will be able to creep at a stress lower than the frictional yield strength of the stronger component. If pressure solution is less efficient, for example because grain size is large, strain rate is high, or rocks are relatively dry, this will be a regime where rates of pressure solution and frictional granular flow are comparable. At such conditions of competition between granular flow and pressure solution at steady state, faults are velocity-weakening and potentially seismogenic [23,80,110–113]. Various combinations of creep and stick-slip will exist depending on spatial and temporal distribution of viscous and frictional deformation, as was discussed in §3b and is illustrated by local through-going faults within a shear zone of competent clasts in a foliated matrix in figure 3*b*. The highest degree of visco-frictional interaction is predicted at depths just below the competent material’s frictional–viscous transition, where both the frictional yield (*τ*_*y*_) and the viscous strength of component material at the imposed strain rate ($\eta_{s}\dot{\gamma }$) are too high for either uniform frictional sliding or viscous flow to occur (figure 3).

### (c) Viscous flow

Deeper than the frictional–viscous transition of the more competent phase, the flow strengths of both materials are less than the frictional yield of the more rigid fault rocks at the steady-state strain rate. Therefore, below the frictional–viscous transition in the competent phase, the bulk flow stress departs significantly from the frictional yield curve. For shear stress to locally reach frictional yield strength at these depths, one of two things must happen; either the stress increases (for example by local stress amplification caused by the flowing matrix; [94,95,101]) or frictional yield decreases (for example by local increase in fluid pressure). In this regime, the viscosity contrast decreases with increasing depth and temperature, and can become very small if both components flow viscously at small driving stresses ($\tau ∼\eta_{s}\dot{\gamma }∼\eta_{w}\dot{\gamma }$; figure 3*c*).

## 5. Modelled effects of rheological heterogeneity on slip behaviour

To further analyse the effects and importance of rheological heterogeneities as described above from the geological record, we take a conceptual approach to modelling two-phase shear zones. Previous numerical studies have also considered effects of heterogeneity on fault zone behaviour. For example, by considering brittle asperities in a viscous matrix representing the seismic–aseismic transition at the base of the subduction megathrust seismogenic zone [114], by alternating velocity-weakening and velocity-strengthening properties in a rate-and-state dependent formulation of fault friction [96,115], or by varying pressure solution kinetics within seismic cycle simulations incorporating a microphysical model [29]. Another approach has been to simulate slow slip transients as initiated by local fracture [116]. Slip rate transients can also arise from interaction between competent clasts in a weak matrix [117], an interaction that also creates local stress amplification [101].

### (a) Method

Using the particle-in-cell finite element code Underworld [118], we compute velocity and pressure fields for a two-dimensional cross section, assuming plane strain, of a shear zone containing more viscous elliptical clasts in a less viscous matrix [97,101]. Both clasts and matrix follow a Newtonian, linear viscosity, but clasts also have a frictional yield strength *τ*_*y*_ dictated by the Mohr–Coulomb failure criterion. This set-up is analogous to matrix deformation by pressure solution, with embedded, higher viscosity clasts that may flow slower than the matrix, or episodically fracture in response to locally amplified stresses or a low frictional yield strength [97]. Dynamic frictional slip is not modelled. All results can be non-dimensionalized and scaled [101], such that the only model variables are the viscosity ratio of the strong and weak components, *η*_*s*_/*η*_*w*_, and the ratio of driving stress to frictional yield *τ*/*τ*_*y*_. Periodic boundary conditions are applied to the left- and right-hand boundaries, the base is kept stationary and a constant driving shear stress, *τ*, is applied to the top boundary. The model domain has a 4 : 1 aspect ratio and a high mesh resolution of 2048 × 512 is used to resolve the anastomosing matrix. The shear zone contains 61% competent clasts, to simulate an anastomosing shear zone system, and these clasts have a power-law size distribution with a constant aspect ratio of 1 : 3 and a maximum long axis of 84% shear zone thickness. We explore how clast fracturing and shear zone strain-rate hetereogeneity depend on varying *η*_*s*_/*η*_*w*_ and *τ*/*τ*_*y*_. Note that because stresses are amplified by the stronger clasts, local stresses will exceed the applied driving stress [101]. Models with *η*_*s*_/*η*_*w*_ = 10^3^ have been described in [101], where more detail on the modelling procedure is available. Additional models with *η*_*s*_/*η*_*w*_ = 10 and 10^2^ were run for this study.

The aim here is not to model specific faults, conditions, scales or settings, but to consider a fault system where interconnected weak zones between more competent lenses may allow strain localization, while we can also assess how deformation is distributed between the two phases and how the fault zone behaves as a bulk aggregate. We are interested in how the ratios *η*_*s*_/*η*_*w*_ and *τ*/*τ*_*y*_ influence the likelihood that a chain of adjacent clasts will have stress states at frictional failure, which is assumed to be analogous to bulk shear zone frictional failure. The former ratio represents the degree of viscous heterogeneity. The latter ratio is also a measure of heterogeneity, because *τ*/*τ*_*y*_ → 1 will mean that the fault zone is essentially at brittle failure, *τ*/*τ*_*y*_ ≪ 1 represents a bulk viscous shear zone, but 0 < *τ*/*τ*_*y*_ < 1 can lead to intermediate behaviours. Considering the ratio *τ*/*τ*_*y*_ is not new—it is an inverse form of the engineering safety factor for slope stability [119], analogous to a flow factor that can control slow versus rapid sediment mass transport [120], and similar ratios describe on-fault frictional deformation versus off-fault ductile matrix flow in experiments [121]. Note that several other factors, such as the clast:matrix ratio, the size, aspect ratio, and size distribution of clasts, and thickness of the shear zone, will also affect the model results—however, these parameters will affect absolute values rather than the general trends, and it is the general trends we wish to discuss here.

### (b) Effects of variable viscosity ratio and *τ*/*τ*_*y*_

**Figure 4.**
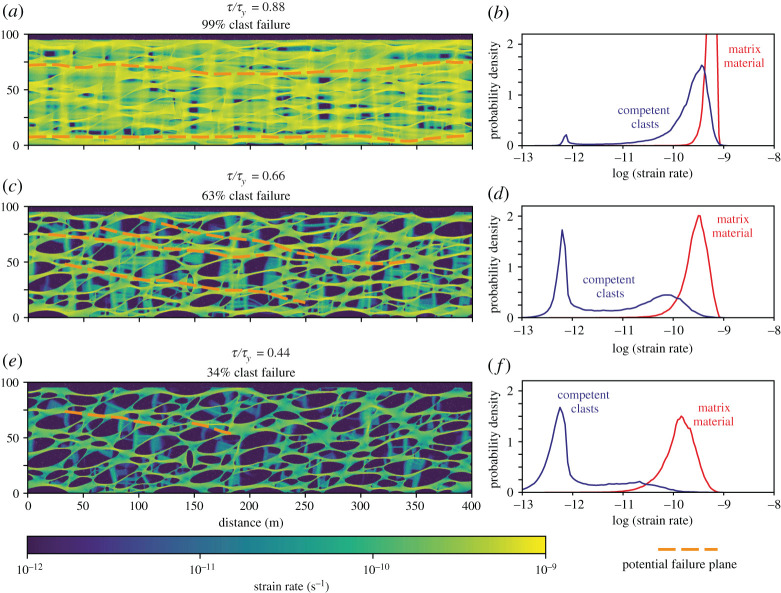
Plots of strain rate distribution within a linear-viscous shear zone, where the viscosity ratio between clasts and matrix is 10^3^. Models are run at different ratios of driving stress (*τ*) to frictional yield stress (*τ*_*y*_) of the competent phase. When *τ*/*τ*_*y*_ approaches 1 (*a*), frictional deformation dominates and allows uniformly high strain rates (*b*). Compare this to a small *τ*/*τ*_*y*_ where only ∼ 1/3 of clast volume is failing frictionally (*e*), and the strain rates are bimodal (*f* ), with much faster deformation in the weak, viscously flowing matrix. In the intermediate case (*c*), sufficient clast volume (greater than 50%) is failing to allow potential for through-going discrete fractures to form locally and transiently, linking fracturing clasts through the matrix material. In this case, the probability density distribution of strain rate (*d*) shows a narrow peak for clasts deforming slowly and viscously, and a broader peak for faster deformation in clasts that are undergoing some level of frictional deformation. The strain rate is given in units of s^−1^ assuming a matrix viscosity of 10^17^ Pa s and *τ*_*y*_ = 160 MPa [97]. (Online version in colour.)

First consider a model with a high viscosity ratio, 10^3^ (figure 4). Such high viscosity ratios are most likely above or slightly below the frictional–viscous transition in the competent phase, in areas of local hydration and alteration to weak minerals, at shallow depths where consolidation is highly variable, or other settings where low viscosity materials encompass competent lenses.

The large viscosity contrast will lead to local strain rate gradients that induce amplified shear stresses in the competent clasts [101], as also predicted for rigid inclusions in both elastic and plastic systems in the absence of clast interaction [122,123]. At driving stress near the frictional yield, *τ*/*τ*_*y*_ = 0.88, this stress amplification is sufficient for 99% of total clast volume to be at frictional failure (figure 4*a*). Because fracturing is now essentially occurring throughout the competent clasts, while the matrix is flowing easily, the aggregate is deforming at a more or less constant strain rate (figure 4*b*). Though dynamic frictional slip is not modelled, we can hypothesize how such slip would be affected by the static stress state calculated here, by considering that earthquake ruptures can potentially propagate through velocity-strengthening patches of similar length scale [115], or that experience a background stress near their frictional yield strength [29], but will likely terminate in regions where the ambient stress is far below the yield strength [124]. In this case, all clasts are at failure and any unstable frictional slip nucleating in one clast may plausibly propagate through the shear zone and a principal slip zone may develop in such a system (figure 4*a*).

At an intermediate *τ*/*τ*_*y*_ of 0.66, 63% of clast material is at frictional yield (figure 4*c*). Under these conditions, the strain rate in the competent clasts covers a broad range, with a peak deforming at a slow strain rate controlled by their viscosity, and others deforming faster as fractures allow them to reach matrix strain rates (figure 4*d*). Though much of the clast stress distribution is lower than the frictional yield, depicted by low strain-rates, hypothesized rupture pathways could still be connected between clasts that are at frictional failure (figure 4*c*) as fractures dynamically overcome shear resistance and velocity-strengthening friction in the matrix. This stress field may therefore be conducive to earthquake nucleation. Note that these potential rupture planes are slightly oblique to shear zone margins and will be limited in length by the shear zone thickness. If *τ*/*τ*_*y*_ is lower still, 0.44, then only 34% of clasts are at frictional failure, and the fault zone is dominated by viscous flow (figure 4*e*); the strain rate distribution is bimodal, with clearly different strain rates in fast flowing matrix and much slower flow in competent clasts (figure 4*f* ). The driving stress is too low, or frictional yield strength too high, for much clast material to be at frictional failure, and the hypothesized failure planes are limited to short segments that rarely connect between multiple clasts without passing through material far from frictional failure (figure 4*c*). Thus, fault lengths are likely to remain shorter than the nucleation length scale for instabilities.

**Figure 5.**
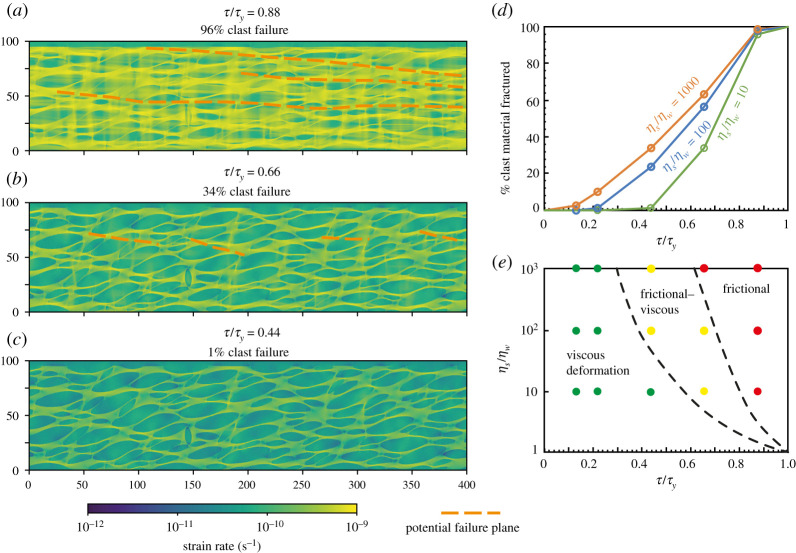
Plots of strain rate distribution within a linear-viscous shear zone where the viscosity ratio between clasts and matrix is 10. As in figure 4, the models are run at different ratios of driving stress (*τ*) to frictional yield strength (*τ*_*y*_) of the competent phase, however, here the viscosity ratio is two orders of magnitude smaller. When *τ*/*τ*_*y*_ approaches 1 (*a*), 96% of clasts are yielding and frictional deformation dominates. There are multiple, potential through-going failure planes with lengths across the model domain. Compare this to a small *τ*/*τ*_*y*_ where here only 1% of clasts are failing frictionally (*c*), as opposed to 1/3 in the models with viscosity ratio of 10^3^. In the intermediate case (*b*), only 34% of clasts are yielding, so that there is limited potential for short, discrete fractures to form, linking only few fracturing clasts through the matrix material. The data plots shown here, those in figure 4 and additional model runs are summarized in (*d*), whereas combined effects of *τ*/*τ*_*y*_ and the viscosity ratio *η*_*s*_/*η*_*w*_ is illustrated in (*e*). In (*e*), points show where models were run, and depict viscous (less than 30% competent material fracturing), frictional–viscous (30–60% competent material fracturing) and frictional (greater than 60% competent material fracturing), respectively. Black dashed lines approximate fields by linear interpolation between calculated points.(Online version in colour.)

A viscosity ratio of 10^3^ in summary gives a range from nearly fully frictional clast deformation if *τ*/*τ*_*y*_ is 0.88, through to dominantly viscous flow at *τ*/*τ*_*y*_ of 0.44, although about 1/3 of clast volume still fails frictionally at this stress ratio, albeit at very limited length scales. Reducing the viscosity ratio to 10 changes the picture to largely remove the intermediate frictional–viscous behaviour (figure 5). In this case, *τ*/*τ*_*y*_ = 0.88 again leads to near total frictional behaviour, with 96% of clast material yielding, and potential for through-going failure planes connecting fracturing clasts across the model domain (figure 5*a*). However, with *τ*/*τ*_*y*_ = 0.66, only 34% of clast material is failing (figure 5*b*), as with *τ*/*τ*_*y*_ = 0.44 if the viscosity ratio is 10^3^. This implies a relatively sharp change from frictional–viscous behaviour with *τ*/*τ*_*y*_ = 0.66 to frictional at *τ*/*τ*_*y*_ = 0.88 (figure 5*a*,*b*), as also indicated by a steepening of the % frictional deformation vs *τ*/*τ*_*y*_ curve in figure 5*d*. Reducing *τ*/*τ*_*y*_ to 0.44 with a viscosity ratio of 10 leaves only 1% of clast material fracturing, i.e. the shear zone is basically entirely viscous (figure 5*c*).

Overall, clast fracturing and therefore visco-frictional interplay increases with increasing viscosity contrast, when 0 < *τ*/*τ*_*y*_ < 1. For a viscosity ratio of 10 the behaviour is uniformly viscous for *τ*/*τ*_*y*_ < 0.4, whereas for viscosity contrasts of 10^2^ and 10^3^, some very minor frictional behaviour is seen at *τ*/*τ*_*y*_ = 0.22 (figure 5*d*). As *η*_*s*_/*η*_*w*_ increases, the transition zone covers a greater range of *τ*/*τ*_*y*_ values (figure 5*e*), because stress concentration in the competent bodies brings them closer to frictional yield [97,101], and the *τ*/*τ*_*y*_ at which frictional deformation ceases to dominate decreases. The transitions between the deformation regimes also occur at lower *τ*/*τ*_*y*_ ratios if *η*_*s*_/*η*_*w*_ increases, because of this stress concentration. The transitions are likely similar at viscosity ratios >10^3^, based on the decreasing spacing of the curves in figure 5*d*.

Deformation style can be characterized by proportion of clast failure, as across the models 34% frictional failure appears to allow local fracturing that can only link between a few clasts (figures 4*e* and 5*b*); 63% clast failure leads to fractures connecting across the bulk or all of the model domain (figure 4*c*). Accordingly, we consider the bulk deformation viscous, and frictional deformation restricted to rare and very local, short length-scale deformation if less than 30% of clast material is yielding. On the other hand, if more than 60% of the clast material is yielding, then through-going fractures accommodating frictional sliding would not need to overcome barriers of clast and/or matrix material far from failure. The intermediate case, 30–60% clast material yielding, can be considered a transitional state where local fractures develop, can link between clasts and clusters of clasts, but are limited in length-scale and rarely span the model domain. These values clearly depend on geometry and clast shape; however, we plot types of deformation in figure 5*e* to demonstrate the shape of the viscous, frictional and frictional–viscous fields that arise from the models in *τ*/*τ*_*y*_ versus *η*_*s*_/*η*_*w*_ space. For the end-member where *η*_*s*_/*η*_*w*_ = 1, the change from frictional to viscous deformation occurs when *τ*/*τ*_*y*_ < 1 and there is no transition zone, because there is no static stress concentration.

## 6. Model application

The models presented above consider a fault zone comprising interconnected viscously weak material surrounding stronger visco-frictional lenses. This geometry can be seen as representative for scales ranging from mineral clasts in gouges, as seen within fault cores or in laboratory experiments, through to anastomosing schistose or phyllonitic shear zones separating relatively intact, km-scale rigid wall rock lenses. Thus, we suggest the conceptual models connecting viscosity contrast and *τ*/*τ*_*y*_ to bulk fault behaviour can be applied to a range of settings and scales. Detailed applications are beyond the scope of this conceptual paper, however, we present some thoughts and a simple illustrative application.

### (a) Depth-dependence of frictional versus viscous deformation

We can use our two-phase numerical models (figures 4 and 5) to explore the dynamics that may control the depth-dependence of the ratio of frictional to viscous behaviour (figure 3). The models were used to predict the degree of visco-frictional interaction, based on the ratios of *η*_*s*_/*η*_*w*_ and *τ*/*τ*_*y*_. These ratios will both change with depth, depending on the depth-dependence of material properties and effective stress state. We will calculate how these ratios are expected to vary for a particular tectonic profile and subsequently map the visco-frictional regimes to depth ranges.

**Figure 6.**
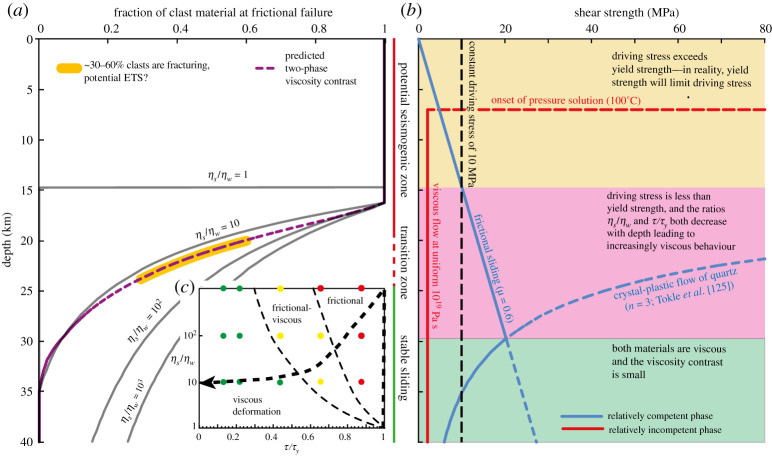
Illustrative example of strength and deformation mode in a two-phase fault zone. In (*a*) the fraction of competent material at frictional yield is plotted against depth for various viscosity contrasts (*η*_*s*_/*η*_*w*_). (*b*) shows the specific example used, which is a simplified subduction thrust, involving a weak phase (red line) with viscosity 10^19^ Pa s deforming by pressure solution at temperatures above 100^°^C, and a competent phase (solid blue line) controlled by Byerlee friction and a quartz flow law [125]. The black dashed line shows a driving stress of 10 MPa. Inset (*c*) depicts the approximate path of subducting rocks with increasing depth along the subduction interface in the deformation style map presented in figure 5*e*.(Online version in colour.)

Conceptually, consider variations in the proportion of competent fault rocks fracturing as a function of depth for a range of viscosity ratios. These are plotted in figure 6*a*, where the solid, grey curves will always have same shape but will be translated shallower or deeper depending on *τ*/*τ*_*y*_. Deeper than a uniformly frictional regime, a transitional zone occurs where the volume fraction of frictional failure decreases with depth. This decrease in fracturing is a result of frictional strength increasing whereas increasingly efficient viscous flow occurs in interconnected incompetent material. As depth increases, viscosity becomes progressively lower relative to a higher frictional yield strength, and at some depth shear is entirely viscous, and any competent material is too frictionally strong relative to bulk strength for any frictional failure to occur. This line of argument, however, assumes a uniform fluid overpressure—it is important to remember that at any depth, if frictional yield can be reduced sufficiently, the proportion of material undergoing frictional failure will increase markedly. Note that this depth variation in deformation is comparable to that well established from geological observations (e.g. figure 3).

### (b) A simple example: the base of the subduction thrust seismogenic zone

We now calculate a specific viscosity contrast profile example, and plot the fraction of competent material undergoing frictional failure against depth (figure 6*a*), based on extrapolating between models as those shown in figures 4 and 5. Mapping viscosities to depth and fraction of material fracturing (dependent on *τ*/*τ*_*y*_; figure 5*d*) is based on subduction-like conditions, including a near-lithostatic pore fluid factor (*λ* = pore fluid pressure/vertical stress) of 0.9, clast friction *μ* = 0.6, low driving stress of 10 MPa, and thermal gradient of 15^°^C km^−1^. As the bulk rheology changes with depth, the constant driving stress is a simplification that implies a varying strain-rate at depth if tectonic slip is approximately uniform. If viscosity decreases with increasing temperature, as expected in a purely viscous regime, then the constant stress assumption implies strain localization and decreasing shear zone width with depth. On the other hand, shear zone width may be buffered by the yield strength of the undeformed wall rocks [126], which corresponds to shear zone widening with depth below the brittle–ductile transition, consistent with figure 1*a*. Such widening would also occur in a visco-frictional shear zone if increasing frictional strength has a greater effect on bulk strength than decreasing viscosity. Separating these models and further assessing the validity of the constant stress assumption could be a subject of future study. We assume the weakest component can deform by pressure solution, with a constant viscosity of 10^19^ Pa s, where *T* ≥ 100^°^C and pressure solution is efficient in quartz; at this temperature, frictional resistance is still less than 10 MPa at the given conditions, and a sharp frictional–viscous transition occurs somewhat deeper for *η*_*s*_/*η*_*w*_ = 1 where *τ*_*y*_ drops below *τ*. The onset of pressure solution will be more gradual than this T-dependent onset of a constant, low viscosity implies, leading to a broader transition zone in reality than in our models. For *η*_*s*_/*η*_*w*_ > 1, total clast failure can occur when *τ*/*τ*_*y*_ < 1, such that the onset of some viscous deformation is seen a few kilometres deeper. Below this, as illustrated also in figures 4 and 5, a relatively sharp change from dominantly frictional to dominantly viscous deformation is seen for *η*_*s*_/*η*_*w*_ = 10. For greater *η*_*s*_/*η*_*w*_, however, local stress amplification leads to a mixed frictional–viscous behaviour over a substantial depth range of up to tens of kilometres.

In a simplified view, the likely heterogeneous subduction thrust comprises weak, phyllosilicate-rich materials representing pelitic sediment and/or altered, hydrated oceanic crust [127], mixed with more competent materials such as sandy or cherty sediment layers and/or basaltic oceanic crust [64]. Although there are certainly depth-variations in the strength of the weak component, over our depths of interest we consider these small compared to competency contrasts, and use a constant, low, Newtonian viscosity to represent pressure-solution creep (figure 6*b*). Consider this weak component mixed with a quartz-dominated rheology, also plotted in (figure 6*b*). This competent quartz rheology is derived based on crystal plastic flow in quartz [125], assuming a constant, relatively slow, clast strain-rate of 10^−13^ s^−1^, such that the clast viscous strength is conservatively underestimated. Then a viscosity ratio can be extracted from these two lithologies and shown as a function of depth (figure 6*a*,*b*).

The viscosity contrast is greatest at the onset of pressure solution, but at this depth, frictional yield is still relatively easy and a major proportion of deformation is therefore frictional (figure 6*a*,*b*). In other words, *τ*/*τ*_*y*_ = 1, and therefore all deformation is frictional independently of viscosity contrast. With increasing depth, frictional yield becomes more difficult, but for *η*_*s*_/*η*_*w*_ > 10^2^, and as long as *τ*/*τ*_*y*_ > ∼0.6 there is likely to still be through-going frictional failure (figure 4). However, as *η*_*s*_/*η*_*w*_ decreases to 10 < *η*_*s*_/*η*_*w*_ < 10^2^ and *τ*/*τ*_*y*_ becomes smaller as *τ*_*y*_ increases, there is a depth range where a fraction of 0.3 to 0.6 of the more competent material is fracturing (figure 6*a*,*c*). By comparison to figures 4 and 5, the top of this depth range may have some through-going frictional planes, but as the competence of the stronger phase decreases, these become shorter and less important in accommodating displacement. Frictional slip propagating from one block at frictional failure and through surrounding matrix would have to overcome the stress deficit of the matrix and/or adjacent blocks far from failure, potentially limiting rupture lengths to the length scale of one block (less than 100 m order; [128]). This may be comparable to the inferred decrease in stress and length-scale of low frequency earthquakes that occur within ETS in Cascadia [129,130], and the mixed behaviour of slow slip and tremor in general—where low frequency earthquakes may represent smaller-scale frictional deformation embedded within a fault deforming aseismically [131]. We make a jump here, assuming our visco-frictional deformation may lead to slow slip; we do not know if that is the case, but it is a tempting comparison given the coincidence between our predicted mixed behaviour and that seen in subduction margins hosting ETS. This comparison is compatible with slow earthquakes as self-driven frictional instabilities that have large nucleation length scales relative to the dimension of the fault hosting them (the potential frictional failure planes within our model shear zone) [132–134]. The potential for such transients to grow into larger instabilities will depend on their stress drop overcoming the shear resistance of the surrounding shear zone, which will be easier if the background stress approaches local fault strength [29]. Observation of increased moment rate during coalescence of slow slip events in Cascadia also implies that interacting instabilities have the potential of overcoming the dampening effect of intervening frictionally stable fault material [135].

The exact depth range where we predict a mixed behaviour with decreasing brittle behaviour with depth depends critically on parameters such as driving stress, fluid pressure and strain rate, but is consistently where the viscosity ratio is at least on the order of 10^1^ to 10^3^, and 0.3 < *τ*/*τ*_*y*_ < 0.6, in the transition zone from frictional to viscous deformation (e.g. figure 6*c*) as is commonly observed. Below this zone, which is likely a very narrow depth range if *η*_*s*_/*η*_*w*_ is 10 or less, *η*_*s*_/*η*_*w*_ approaches one and *τ*/*τ*_*y*_ approaches zero, such that viscous creep dominates unless local conditions, likely very high fluid pressures, allows very local frictional failure. Such local, potential instabilities will be dampened by surrounding viscous material and suppressed by a large nucleation length scale; however, unstable slip may occur if fault weakening by fluid pressurization overcomes velocity strengthening [136]—and this has been suggested previously as a mechanism for ETS spatially separated from the frictional–viscous transition [102].

We focused here on the base of the subduction interface seismogenic zone. If aseismic slip at the updip end of the seismogenic zone also involves some component of viscous deformation, then our assumption of a sharp, *T*-dependent onset of pressure solution at 100^°^C hides any shallower, near-trench effects of mixed frictional–viscous or seismic–aseismic deformation. Such shallow, visco-frictional deformation may occur in some subduction zones, particularly where carbonates make up a considerable proportion of the incoming sediment sequence [137,138]. We also recall that within the shallow, dominantly frictional regime, variations in consolidation, porosity, fluid pressure or material properties in time and space may add further heterogeneity than what we model here, even in the absence of a viscous component [47]. We highlight the shallow portion of subduction zones as a target for future study.

## 7. Is complex fault zone behaviour a reflection of rheological heterogeneity?

There appears to be a broad agreement among structural geologists that fault zone heterogeneity gives rise to a range of deformation styles, and a common co-existence of brittle and ductile structures [48,54,55,69,77,80,91–93,98,128]. However, because heterogeneity of some magnitude is present at some scale in just about every rock outcrop, a question remains of how to quantify heterogeneity, and to what extent the rheological heterogeneity inferred from the rock record reflects the heterogeneity in slip style observed by geophysical techniques in active fault zones [5,40,41,43,44,131]. One approach is to consider heterogeneity as the strength ratio between co-existing materials, and their geometrical distribution [77,91]. This, however, has limitations. If the stronger phase is very far from failure and not forming an interconnected network, it will accommodate little deformation and the weaker phase controls rheology. Similarly, if the stronger phase is very close to failure and there is insufficient interconnected weak material for the strong phase to be rheologically insignificant, it likely controls rheology, with fractures propagating though the weaker phase however easily it may deform viscously. These effects have been considered before [77,89,91], and were illustrated here in (figure 5*e*). There is also the case of a competent phase close to failure, but in low volumetric proportions. Rupture propagation is then limited if the volume of matrix in between blocks is too large and experiencing a background stress far below its frictional yield [29,96,115]. We add here, that although one competent block is at failure, the adjacent one might not be, depending on stress heterogeneity or local weakening by, for example, fluid pressure. Stress heterogeneity and local stress amplification is also reduced at small fractions of competent material [101], and thus the frictional–viscous regime may be narrow if weak, relatively viscous materials dominate volumetrically. These potential heterogeneities in *τ*/*τ*_*y*_ are analogous to frictional variation providing barriers in dynamic rupture models of rough faults [139,140]—in that a variable frictional yield will make the fault closer to failure in some areas compared to others. In our model, the barriers effectively disappear when either the driving stress or pore pressure is very high (*τ*/*τ*_*y*_ → 1), as also occurs in the rupture models.

Our models in figures 4 and 5 have a high clast/matrix volume ratio (61%) such that they best represent an anastomosing shear zone network encompassing relatively high viscosity lenses. From a range of geological observations, we suggest that heterogeneity varies with depth, and arises from a range of processes including diagenesis, the degree to which pressure solution accommodates creep in the seismogenic zone, and the strain rates that can be accommodated by diffusion and dislocation creep in different materials. We suggest that the two basic, general, controls on bulk deformation are the contrast in deformation behaviour between co-existing fault rocks, and how far the bulk driving stress is from frictional yield. Strength contrast alone is not a sufficient measure of heterogeneity, if the aim is to determine the bulk frictional–viscous deformation style of a fault or shear zone. For our models, viscosity ratios of 10 and 1000 give depth ranges 3 and 10 km wide, respectively, where *τ*/*τ*_*y*_ ratios are high and frictional and viscous deformation modes co-occur as a result of stress amplification in the stronger phase, independently of the source of heterogeneity. This depth range of visco-frictional behaviour can be shifted up/down dynamically, depending on *τ* and/or fluid pressure, which may both vary substantially. Observed variations in depths of SSEs and LFEs, deemed representative of a transitional regime, may be determined by these stress and fluid pressure variations, without having to invoke variation in rheology or geothermal gradient.

## 8. Conclusion

Although geological and rheological heterogeneity is pointed out in an increasing array of fault zones in several tectonic settings, the magnitude and length scale of such heterogeneity varies. To what extent can ’heterogeneity’ in material properties be measured, quantified and tied to heterogeneity in fault zone deformation? We propose that, at the first order, fault slip style in a heterogeneous tabular fault zone is controlled by a combination of viscosity contrast and the ratio, *τ*/*τ*_*y*_, of bulk driving stress to frictional yield strength.

In this conceptual model, earthquakes require the frictional yield to be reached, and steady viscous flow requires conditions far from the frictional yield, independently of fault zone viscosity contrasts. Intermediate slip speeds may arise when driving stress is sufficient to arise local frictional failure by stress amplification, but this failure is limited in length-scale by surrounding viscously deforming media and heterogeneity of clast stresses (and/or strengths). The conditions where this is likely cover a larger range of *τ*/*τ*_*y*_ at greater viscosity contrast.

Frictional deformation is more difficult and reliant upon local stress concentrations near the frictional–viscous transition, and this is consequently also where the models presented here predict a zone of intermediate and mixed deformation style. At greater depths, viscosity ratios are small and driving stress far from frictional yield, such that frictional sliding and mixed transitional behaviour is unlikely, but possible in the presence of high to extreme local fluid overpressures.

Considering the variation in fault slip style with depth in some subduction and strike slip zones, and the numerical models presented here, intermediate fault slip speeds as seen in tremor and slow slip may correspond to slip in highly heterogeneous stress fields that may arise from *τ*/*τ*_*y*_ ratios that are intermediate between low values suppressing frictional yield and high values allowing through-going frictional failure. The exact values depend on viscosity contrast, but lead to fractures limited by viscous, weak matrix but locally allowed to nucleate by an ability for more competent lenses to flow or fracture. This is easier over a larger depth range at higher viscosity contrast, as otherwise, the bulk rheology will tend towards either fracture or viscous flow.

## Data accessibility

The open-source geodynamic code Underworld is available at http://www.underworldcode.org, and model parameters required to replicate the results are detailed in the manuscript and in [97].
